# Chemo-Enzymatic Synthesis of a Multi-Useful Chiral Building Block Molecule for the Synthesis of Medicinal Compounds

**DOI:** 10.3390/molecules16086747

**Published:** 2011-08-09

**Authors:** Toshiki Nakano, Yusuke Yagi, Mizuki Miyahara, Akio Kaminura, Motoi Kawatsura, Toshiyuki Itoh

**Affiliations:** 1 Department of Applied Molecular Bioscience, Graduate School of Medicine, Yamaguchi University, 2-16-1 Tokiwadai, Ube 755-8611, Japan; 2 Department of Chemistry and Biotechnology, Graduate School of Engineering, Tottori University, 4-101 Koyama-minami, Tottori 680-8552, Japan

**Keywords:** nitro alcohol, quaternary chiral carbon, lipase, optically active, synthesis

## Abstract

Optical resolution of 2-methyl-2-nitrobut-3-en-1-ol has been accomplished using a “low-temperature lipase-catalyzed transesterification” carried out at −40 °C.

## 1. Introduction

**Scheme 1:**
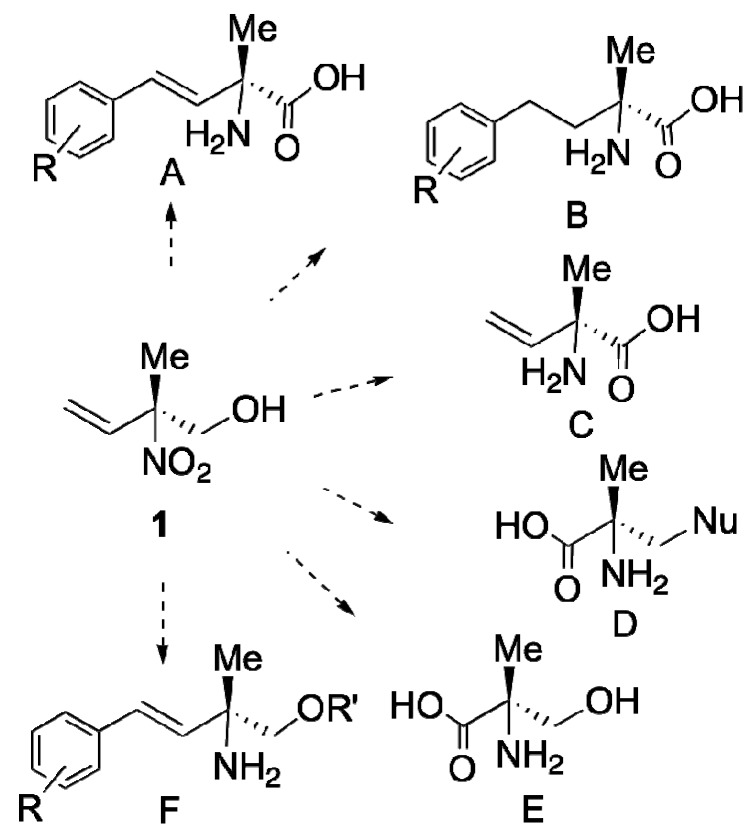
Multi-useful chiral building block for the synthesis of non natural amino acids and amino alcohols.

Chemo-enzymatic reaction protocols are now well recognized as a very useful means to prepare optically active compounds [1,2,3]. 2-Methyl-2-nitrobut-3-en-1-ol (±**1**) was prepared by a simple method using the nitroaldol reaction for nitroalkenes [4], and it has been expected to become a useful building block for the synthesis of various types of non-natural amino acids (A ~ E) or amino alcohols (F), as illustrated in Scheme 1. However, no one has yet succeeded in preparing optically pure nitro alcohol **1**, so preparation of optically active **1** using a practical protocol has thus been strongly desired.

Lipases have wide applicability for various types of substrates [1,2,3,5], however, it is generally not easy to use the lipase-mediated reaction for the kinetic resolution of a primary alcohol like alcohol **1**, because the chiral carbon is remote from the reaction point in such a type of compound [2]. Since preparation of chiral compounds that have a quaternary stereocenter is an important challenge for modern organic synthesis, several examples have been demonstrated using enzymatic reactions [6,7,8,9,10,11,12,13]. Herein, we report the establishment of a protocol that affords both enantiomers of 2-methyl-2-nitrobut-3-en-1-ol (**1**) using a lipase-catalyzed reaction; the “low-temperature lipase-catalyzed reaction” protocol [14,15,16] was shown to be the key technology to accomplish the desired reaction with sufficient enantioselectivity.

## 2. Results and Discussion

**Scheme 2:**
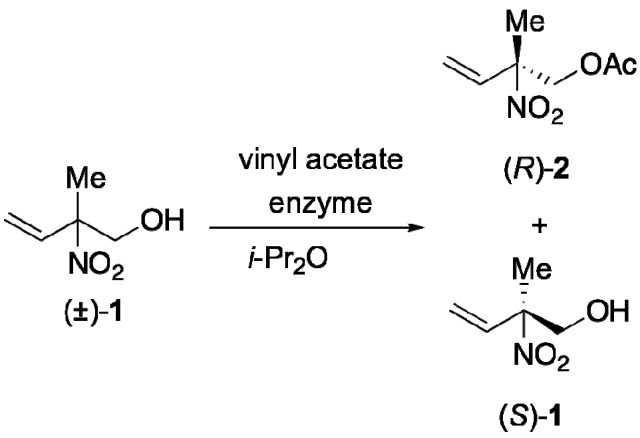
Kinetic resolution of (±)-**1** using an enzymatic reaction.

We initially attempted to resolve (±)-**1** *via* lipase-catalyzed transesterification using vinyl acetate as acyl donor in diisopropyl ether (*i*-Pr_2_O) under standard reaction temperature at 35 °C (Scheme 2); however, after evaluating commercial lipases, we soon recognized that it would be a very tough task for us to find a suitable enzyme, as we were unsuccessful in finding an appropriate enzyme that could convert **1** to the corresponding acetate with acceptable enantioselectivity. Among the 17 types of commercial enzymes tested, only five lipases PS, SL-25, PL, Novozyme 435 and QLM gave the corresponding acetate, but all with insufficient enantioselectivity. Although lipases QLM and SL-25 gave somewhat better results, the E values [17] of the reactions were 7.2 (QLM) and 6.0 (SL-25), respectively. Since lipase QLM gave the best E value, we next attempted to optimize the reaction condition using lipase QLM as a catalyst.

It has long been believed that enantioselectivity of lipase-catalyzed reaction could be explained by the traditional three point attachment rule [2,18]. According to this rule, optimization of a substrate structure or protein engineering of lipases might be essential to control the enantioselectivity of the enzymatic reactions [2].

On the other hand, Ema *et al*. proposed that the enantioselectivity of the lipase-catalyzed reaction might be determined mainly by kinetic preference due to the conformational requirements and repulsive interaction on the transition state [19,20]. The model allows enhancement of the enantioselectivity of lipase-catalyzed reaction simply by changing the reaction temperature. Sakai and co-workers, in fact, demonstrated that efficient kinetic resolution of primary alcohols was realized using the “low-temperature transesterification method” [14,15,16]. Sakai showed that lipase-catalyzed transesterification of (2,2-dimethyl-1,3-dioxolan-4-yl)methanol in *i*-Pr_2_O proceeded even at −40 °C; the E value of the reaction at 30 °C was just 9, while it reached 55 when the reaction was conducted at −40 °C [16]. Therefore we decided to apply “the low-temperature method” to our lipase-catalyzed reaction (Scheme 1, and the results are summarized in Table 1).

**Table 1:** Results of lipase QLM-catalyzed transesterification of (±)-**1**.

Entry	Temp	Time	% *ee* of acetate (*R*)-2 ^a^(% yield)	% *ee* of alcohol (*S*)-1 ^b^ (% yield)	% conv.	E value ^c^
1	35	10 min.	54 (35)	75 (28)	58	7.2
2	20	25 min.	66 (24)	35 (53)	35	6.8
3	0	25 min.	67 (37)	42 (50)	39	7.6
4	−20	30 min.	76 (21)	24 (53)	24	9.3
5	−40	25 min.	83 (22)	32 (69)	28	15
6	−40	26 h	58 (44)	94 (27)	64	13

^a^ Determined by HPLC analysis using CHIRALCEL OB-H, hexane/*i*-PrOH = 19/1, 0.5 mL/min; ^b^ Determined by HPLC analysis using CHIRALCEL AD-H, hexane/*i*-PrOH = 19/1, 0.5 mL/min. ^c^ E value was calculated by % *ee* of (*R*)-**2** (*ee_p_*) and % *ee* of (*S*)-**1** (*ee_s_*). E= ln[(1 − ^c^ (1 + *ee_p_*)) / ln[(1 – c (1 − *ee_p_*)); here, c means conv. which was calculated by the following formula according to reference [17]: c = *ee_s_* / (*ee_p_* + *ee_s_*).

**Scheme 3:**
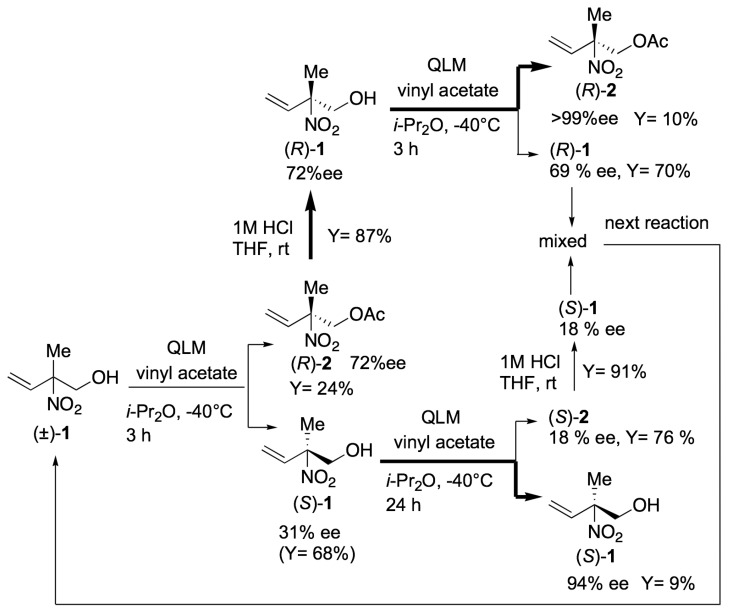
Protocol of preparation of chiral **1** using “low-temperature lipase-catalyzed transesterification”.

Lipase QLM-catalyzed transesterification of (±)-**1** proceeded very rapidly, and we obtained acetate (*R*)-**2** in 35% yield with 54% *ee*, and unreacted alcohol (*S*)-**1** was recovered from the reaction mixture in 28% yield with 75% *ee* after just 10 min of reaction (entry 1). Enantiomeric excess of the product and unreacted substrate were determined by HPLC analysis using a chiral column. A slightly enhanced enantioselectivity was recorded when the reaction was carried out at −20 °C (entry 4), and it finally reached E = 15 at −40 °C (entry 5). Since the reaction rate was very fast, we obtained (*R*)-**2** with 83% *ee* when the reaction was stopped at 25 min (entry 5), while 94% *ee* of (*S*)-**1** was obtained after 26 h of reactions (entry 6); no improved enantioselectivity was recorded when the reaction was conducted at −60 °C. Based on the results, we have developed a protocol providing (*R*)-**2** and (*S*)-**1** with high enantiomeric purities as illustrated in Scheme 3.

Racemic (±)-**1** was subjected to lipase-QLM-catalyzed transesterification at −40 °C, after being stirred for 3 h, the reaction was stopped and the acetate (*R*)-**2** (72% *ee*) and alcohol (*S*)-**1** (31% *ee*) were separated. Enantiomeric purities of (*R*)-**2** and (*S*)-**1** were not sufficient at this stage, so (*R*)-**2** was converted to (*R*)-**1** by acid hydrolysis in 87% yield without any loss of the optical purity. The resulting 72% *ee* of alcohol (*R*)-**1** was subjected to a second transesterification. After 3 h reaction, optically pure (*R*)-**2** (>99% *ee*) was obtained in 10% yield (the upper route in Scheme 3). (*S*)-**1** (31% *ee*) was subjected to a second reaction for 24 h and 94% *ee* of (*S*)-**1** was obtained in 9% yield (the bottom route in Scheme 3). Although the chemical yield of each reaction was insufficient, this protocol made it possible to provide (*R*)-**2** and (*S*)-**1** with high optical purity. After repeating the process, we succeeded in obtaining multiple grams of (*R*)-**2** and (*S*)-**1** with excellent optical purity (Scheme 3).

Development of efficient means for preparing chiral compounds that have a quaternary chiral center has been a challenging area in the field of synthetic organic chemistry. In particular, it is difficult to achieve this aim by enzymatic reaction because hydrolytic enzymes are usually unable to accept sterically hindered substrates bearing fully substituted quaternary carbons [2]. It should be emphasized that the present “low-temperature lipase-catalyzed reaction” provides a possible solution to this problem.

## 3. Experimental

### 3.1. General Procedures

Reagents and solvents were purchased from common commercial sources and used as received or purified by distillation over appropriate drying agents. Reactions requiring anhydrous conditions were carried out under dry argon, freshly distilled solvents, and magnetic stirring. We tested the following commercial lipases: Lipase QL and QLM (*Alcaligenes* sp., Meito), Lipase SL and SL-25 (*Burlholderia* *cepacia*, Meito*)*, Novozyme 435 (*Candida antarctica*, NOVO), Lipase OF *(Candida rugosa* lipase, Meito), Lipase PS (*Burlholderia* *cepacia*, Amano), Lipase AL (*Acromobacter* sp., Meito), Lipase PL (*Alcaligenes sp*, Meito), Lipase A (*Aspergillus niger*, Amano), Lipase AK (*Pseudomonas fluorescens*, Amano), Lipase D (*Actinomadura* sp., Meito), Lipase MY (*Candida cylindracea*, Amano), Lipase F-AP15 (*Rhizopus oryzae*, Amano), Lipase AY (*Pseudomonas fluorescence)*, Lipase TL (*Pseudomonas stutzeri*, Amano), and PPL (Porcine pancreatin lipase, Sigma). Thin layer chromatography was performed with the indicated solvents and Wako gel B-5F. ^1^H-NMR spectra was recorded on JEOL (500 MHz). ^13^C-NMR spectra was recorded on JEOL (125, 100MHz). Chemical shifts are expressed in ppm downfield from tetramethylsilane (TMS) in CDCl_3_ as an internal reference. Optical rotation was measured with a JASCO DIP-370 digital polarimeter. The optical purity was determined by HPLC analysis using CHIRALCEL OB-H and AD-H (Daicel). 

### 3.2. Preparation of 2-methyl-2-nitrobut-3-en-1-ol *(±1) (Scheme 4) [4]*

Nitroethane (22.6 g, 301 mmol) was reacted with acetaldehyde (25.5 mL, 451 mol) in the presence of 1,8-diazabicyclo[5.4.0]undec-7-ene (DBU) (2.0 mL, 13.3 mmol) at 0 °C and the mixture was stirred for 2 h at rt. The reaction was quenched by addition of 10 mL of 1 M HCl aqueous solution and 100 mL of diethyl ether, then the organic layer was washed with 1 M HCl (3 times) and brine (3 times) and dried over MgSO_4_. After evaporation, Kugelrohr distillation of the resulting oil gave 3-nitrobut-2-ol (**3**) (9.24 g, 77.6 mmol) in 26% yield. Nitroalcohol **3** (9.24 g, 77.6 mmol) was treated with acetic anhydride (7.8 mL, 82.5 mmol) in the presence of 5 drops of concd. sulfuric acid and the mixture was stirred at rt for 4 h. To this mixture was added 100 mL of diethyl ether and the resulting organic layer was washed with brine (5 times) and dried over NaSO_4_. After removal of the solvent by evaporation, the resulting oily product was mixed with sodium acetate (6.49 g, 79.1 mmol) and the acetate removed under reduced pressure at 1.0 Torr at 100 °C. After being cooled to rt, the resulting product was diluted with hexane (100 mL) and the organic layer was washed with water (3 times) and dried over NaSO_4_. Kugelrohr distillation gave 2-nitrobut-2-ene (**4**) (3.11 g, 30.8 mmol) in 40% yield. To an acetonitrile (100 mL) solution of **4** (1.10 mg, 10 mmol) was added formaldehyde (1.41 g, 16.4 mmol) and 1,4-diazabicyclo[2.2.2]octane (DABCO) (150 mg, 1.3 mmol) and the mixture was stirred at rt for 24 h, then 1 M HCl (10 mL) and water (50 mL) were added. The mixture was extracted with ethyl acetate and the combined organic layer was washed with brine (3 times) and dried over NaSO_4_; evaporation and silica gel flash column chromatography (hexane: ethyl acetate = 10:1 to 5:1) then afforded (±)-**1** (1.00 g, 8.30 mmol) in 76% yield. ^1^H-NMR (500 MHz, δ, CDCl_3_): 6.17–6.11 (1H, dd, *J* = 17.1 Hz, 6.3 Hz), 5.49 (1H, d, *J* = 17.1 Hz), 5.43 (1H, d, *J* = 6.3 Hz), 4.12–4.09 (1H, m), 3.82–3.80 (1H, m), 2.54 (1H, OH, brs) 1.73 (3H, s); ^13^C-NMR (125 MHz, δ, CDCl_3_) 134.2, 119.0, 92.0, 67.5, 19.5; IR (neat, cm^−1^) 3415, 3098, 2997, 2881, 2946, 1732, 1545, 1461, 1419, 1380, 1349, 1059, 944.

**Scheme 4:**
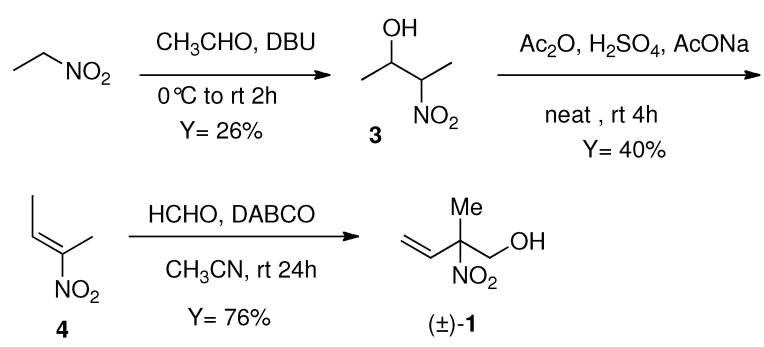
2-methyl-2-nitrobut-3-en-ol (±)-**1**.

### 3.3. Lipase-Catalyzed Transesterification

To a mixture of (±)-**1** [4] (5.00 g, 38.2 mmol) and vinyl acetate (5.2 mL, 57 mmol) in *i*-Pr_2_O (200 mL) was added lipase QLM powder (2.5 g) and the mixture was stirred at −40 °C. After stirring for 3 h, the reaction mixture was filtered through a glass sintered filter with a Celite pad to remove the enzyme. The filtrate was evaporated and chromatographed on a silica gel flash column (hexane: ethyl acetate = 10:1 to 5:1) to give (*R*)-**2** (1.59 g, 9.17 mmol, 24%, 72% *ee*) and (*S*)-**1** (3.40 g, 26.0 mmol, 68%, 31% *ee*). Optical purity was determined by HPLC analysis on a chiral column. For (*R*)-**2**: (CHIRALCEL OB-H, Daicel), hexane/*i*-PrOH = 19:1, 0.5 mL/ min. R_t_ of (*R*)-**2** = 22.8 min; R_t_ of (*S*)-**2** = 25.7 min.; For (*S*)-**1**: (CHIRALCEL AD-H, Daicel), hexane/*i*-PrOH = 19:1, 0.5 mL/ min. R_t_ of (*R*)-**1** = 23.9 min; R_t_ of (*S*)-**1** = 27.0 min. (*R*)-**2**: ^1^H-NMR (500 MHz, δ, CDCl_3_) 6.17–6.12 (1H, dd, *J* = 17.2 Hz, 6.3 Hz), 5.48 (1H, d, *J* = 15.3 Hz), 5.45 (1H, d, *J* = 5.0 Hz), 4.58 (1H, d, *J* = 11.5 Hz), 4.40 (1H, d, *J* = 12.0 Hz), 2.01 (3H, s), 1.73 (3H, s); ^13^C-NMR (125 MHz, δ, CDCl_3_) 169.9, 133.6, 119.4 89.2, 67.3, 20.5, 19.5; IR (neat, cm^−1^) 3000, 2955, 2887, 1753, 1550, 1231, 1051. The absolute configuration of (*R*)-**1** was confirmed by comparison with the sign of specific rotation value of (*R*)-2-amino-2-methyl-4-(4-(heptyloxy)phenyl)butan-1-ol (**5**) [21,22] ([α]_D_ −14.4 (c 0.03, CHCl_3_), lit [21] −14.0 (CHCl_3_)) which was derived from our compound (*R*)-**1** (96% *ee*). The results agree with the established enantio-favoritism of lipase QL-catalyzed transesterification [23].

### 3.4. Synthesis of *(R)*-2-Amino-2-methyl-4-(4-(heptyloxy)phenyl)butan-1-ol *((*R*)-**5**) (Scheme 5)*

*(*R*)-benzyl (2-methyl-2-nitrobut-3-en-1-yl) carbonate ((R)-6):* DMAP (2.45 g, 20.06 mmol) and *i*Pr_2_NEt (5.1 mL, 30.0 mmol) were added to a solution of (*R*)-**1** (2.62 g, 19.98 mmol, 96% *ee*) in CH_2_Cl_2_ (30 mL). A solution of cbz-Cl (3.5 mL, 24.5 mmol) in CH_2_Cl_2_ (10 mL) was added to the solution dropwise. The mixture was stirred at room temperature for 48 h, then poured into 1 M HCl (20 mL). The organic layer was separated and the water layer was extracted with CH_2_Cl_2_ (3 × 30 mL). The organic phases were combined and dried over Na_2_SO_4_. After filtration, the filtrate was concentrated and the residue was purified by flash chromatography (hexane-EtOAc 3:1) to give (*R*)-**6** in 95% yield (5.05 g) Oil. The enantiomeric excess of (*R*)-**6** was determined by HPLC analysis, t_R_ 23.9 min ((*R*)-**6**), t_R_ 27.0 min ((*S*)-**6**) [CHIRALCEL AD-H (0.46 cm × 25 cm) (from Daicel Chemical Ind., Ltd.) hexane/*i*-PrOH, 95/5, 0.5 mL/min] as 96% *ee*: [α]_D_ +24.8 (c 1.67, CHCl_3_); ^1^H-NMR (500 MHz, δ, CDCl_3_) 7.39–7.34 (m, 5H), 6.11 (dd, *J* = 17.4, 10.9 Hz, 1H), 5.46 (d, *J* = 10.9 Hz, 1H), 5.46 (d, *J* = 17.4 Hz, 1H), 5.16 (s, 2H), 4.69 (d, *J* = 11.7 Hz, 1H), 4.42 (d, *J* = 11.7 Hz, 1H), 1.75 (s, 3H); ^13^C-NMR (126 MHz, δ, CDCl_3_) 154.43, 134.79, 133.54, 128.87, 128.77, 128.55, 120.00, 89.18, 70.52, 70.35, 19.61; Anal. Calcd. for C_13_H_15_NO_5_: C, 58.86; H, 5.70; N, 5.28. Found: C, 58.75; H, 5.77; N, 5.26.

(R,E)*-benzyl (4-(4-(heptyloxy)phenyl)-2-methyl-2-nitrobut-3-en-1-yl) carbonate* ((*R*)-**8**): A solution of (*R*)**-6** (200.0 mg, 0.754 mmol) in dry CH_3_CN (4 mL) was purged by N_2_ and NaOAc (188.0 mg, 2.292 mmol), Pd_2_dba_3_ (35.0 mg, 0.038 mmol) and *p*-C_7_H_15_OC_6_H_4_N_2_BF_4_ (**7**) [24] (464.0 mg, 1.516 mmol) were added. The resulting mixture was stirred at room temperature for 48 h. The reaction mixture was concentrated and the residue was subjected to flash chromatography (silica gel/hexane-EtOAc 12:1 then 10:1 then 6:1) to give (*R*)-**8** in 63% yield (216.0 mg). [α]_D_ +80.2 (c 0.46, CHCl_3_). ^1^H-NMR (500 MHz, δ, CDCl_3_) 7.41–7.35 (m, 5H), 7.32 (d, *J* = 8.3 Hz, 2H), 6.86 (d, *J* = 8.2 Hz, 2H), 6.70 (d, *J* = 16.0 Hz, 1H), 6.24 (d, *J* = 16.8 Hz, 1H), 5.17 (s, 2H), 4.80 (d, *J* = 11.3 Hz, 1H), 4.48 (d, *J* = 11.0 Hz, 1H), 3.96 (t, *J* = 6.6 Hz, 2H), 1.85 (s, 3H), 1.82–1.75 (m, 2H), 1.51–1.25 (m, 8H), 0.90 (t, *J* = 6.8 Hz, 3H); ^13^C-NMR (126 MHz, δ, CDCl_3_) 160.10, 154.54, 134.27, 128.86, 128.78, 128.56, 128.48, 121.48, 114.83, 89.30, 70.90, 70.30, 68.16, 31.80, 29.22, 29.08, 26.00, 22.63, 19.89, 14.12; HRMS (ESI M+H) *m/z* 456.2404. Calcd for C_26_H_34_NO_5_ 456.2386.

*Preparation of* (R)*-2-amino-2-methyl-4-(4-(heptyloxy)phenyl)butan-1-ol* ((*R*)-**5**): (*R*)**-8** (199 mg, 0.0437 mmol) was dissolved in MeOH (3 mL) and Pd-C (10%, 20 mg) was added. The mixture was placed autoclave and stirred at room temperature under hydrogen atomosphere at 5 MPa for 50 h. After filtration, the filtrate was concentrated to give (*R*)**-5** (130 mg) in 97% yield. Absolute configuration of (*R*)-**5** was confirmed by comparison with the sign of specific rotation value of that reported: ([α]_D_ −14.4 (c0.03, CHCl_3_), lit [21] −14). ^1^H-NMR (500 MHz, δ, CD_3_OD) 7.10 (d, *J* = 8.5 Hz, 2H), 6.80 (d, *J* = 8.6 Hz, 2H), 3.92 (t, *J* = 6.4 Hz, 2H), 3.39 (d, *J* = 10.6 Hz, 1H), 3.36 (d, *J* = 10.9 Hz, 1H), 2.57 (ddd, *J* = 2.4, 7.9, 10.2 Hz, 2H), 1.81–1.69 (m, 2H), 1.69–1.58 (m, 2H), 1.52–1.42 (m, 2H), 1.42–1.27 (m, 6H), 1.09 (s, 3H), 0.91 (t, *J* = 6.4 Hz, 3H); ^13^C-NMR (126 MHz, δ, CD_3_OD) 157.36, 134.55, 128.85, 114.12, 69.52, 67.65, 52.42, 41.22, 31.70, 29.19, 29.01, 28.94, 25.86, 22.78, 22.38, 13.15; HRMS (ESI M+H) *m/z* 413.1642. Calcd for C_23_H_27_NO_4_S 413.1661.

**Scheme 5:**
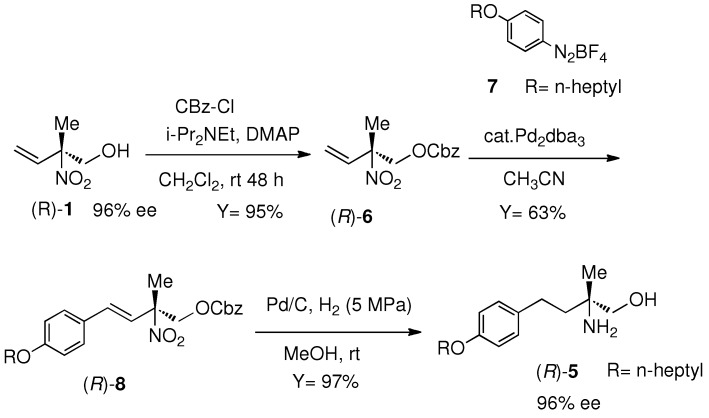
(*R*)-2-amino-2-methyl-4-(4-(heptyloxy)phenyl)butan-1-ol (**5**).

### 3.5. Conversion of (R)-2 to (R)-1 by Acid Hydrolysis

To a THF (3.0 mL) solution of (*R*)-**2** (250 mg, 1.44 mmol) was added 1 M aqueous HCl solution (3.0 mL) at rt and the mixture was stirred for 3 h at rt, and then concd. HCl (3.0 mL) was added and the mixture was further stirred for 72 h at rt. The reaction mixture was neutralized carefully with saturated sodium bicarbonate aqueous solution and extracted with dichloromethane. The combined organic layer was dried under MgSO_4_ and the solvent removed by evaporation. (*R*)-**1** (164 mg, 1.25 mmol) was obtained in 87% yield after silica gel flash column chromatography (hexane: ethyl acetate = 10:1 to 5:1).

### 3.6. Preparation of Optically Pure (R)-2 (The Upper Route in Scheme 2)

To a mixture of (*R*)-**1** (72% *ee*, 1.50 g, 8.67 mmol) and vinyl acetate (1.0 mL, 13 mmol) in *i*-Pr_2_O (60 mL) was added lipase QLM powder (0.75 g) and the mixture was stirred at −40 °C. After being stirred for 3 h, the reaction mixture was filtered through a glass sintered filter with a Celite pad to remove the enzyme. The filtrate was evaporated and chromatographed on a silica gel flash column (hexane: ethyl acetate = 10:1 to 5:1) to give (*R*)-**2** (150.6 mg, 0.870 mmol, 10%, >99% *ee*) and (*R*)-**1** (1.05 g, 6.07 mmol, 70%, 69% *ee*): (*R*)-**2**: [α]^26^_D_ −16.5 (c 0.57, CDCl_3_), >99% *ee*.

### 3.7. Preparation of (S)-***1*** with High Optical Purity (Bottom Route in Scheme 3)

To a mixture of (*S*)-**1** (31% *ee*, 3.40 g, 25.9 mmol) and vinyl acetate (3.5 mL, 39 mmol) in *i*-Pr_2_O (120 mL) of was added lipase QLM powder (1.70 g) and the mixture was stirred at −40 °C. After being stirred for 24 h, the reaction mixture was filtered through a glass sintered filter with a Celite pad to remove the enzyme. The filtrate was evaporated and chromatographed on a silica gel flash column (hexane: ethyl acetate = 10:1 to 5:1) to give (*S*)-**2** (3.41 g, 19.6 mmol, 76%, 18% *ee*) and (*S)*-**1** (305.5 mg, 2.33 mmol, 9%, 94% *ee*): (*S*)-**1**: [α]^26^_D_ +76.8 (c 0.26, CDCl_3_), 94% *ee*. (*S*)-**2** was converted to (*S*)-**1** (18% *ee*) by acid hydrolysis and the resulting (*S*)-**1** (2.34 g) was combined with (*R*)-**1** (1.05 g), which was obtained by the upper route in Scheme 2, to prepare low % *ee* substrate alcohol **1** (3.39 g, 7.4% *ee* (*R*)), then used for the next cycle of the lipase-catalyzed reaction.

## 4. Conclusions

In summary, we established a convenient protocol to prepare both enantiomers of 2-methyl-2-nitrobut-3-en-1-ol (**1**) with over 94% *ee* using lipase-catalyzed transesterification under low temperature reaction conditions. It was possible to apply the reaction protocol to the multi gram scale preparation and we succeeded in preparing the desired compounds easily. Synthetic application of a medicinal compound using optically active **1** is now ongoing in our laboratory.
